# Unraveling Host-Gut Microbiota Dialogue and Its Impact on Cholesterol Levels

**DOI:** 10.3389/fphar.2020.00278

**Published:** 2020-04-03

**Authors:** Remy Villette, Pukar KC, Sophie Beliard, Maria Fernanda Salas Tapia, Dominique Rainteau, Maryse Guerin, Philippe Lesnik

**Affiliations:** ^1^ INSERM, UMRS U1166, “Integrative Biology of Atherosclerosis” and Sorbonne Université, Paris, France; ^2^ Aix-Marseille Université, INSERM U1263, INRA, C2VN, Marseille, France; ^3^ APHM, La Conception Hospital, Marseille, France; ^4^ Sorbonne Université, Inserm, Centre de Recherche Saint-Antoine, AP-HP, Hôpital Saint Antoine, Département de Métabolomique Clinique, Paris, France

**Keywords:** gut microbiota, microbiome, gut metabolites, cholesterol, LDL-cholesterol, cholesterolemia, dyslipidemia and cardiovascular disease

## Abstract

Disruption in cholesterol metabolism, particularly hypercholesterolemia, is a significant cause of atherosclerotic cardiovascular disease. Large interindividual variations in plasma cholesterol levels are traditionally related to genetic factors, and the remaining portion of their variance is accredited to environmental factors. In recent years, the essential role played by intestinal microbiota in human health and diseases has emerged. The gut microbiota is currently viewed as a fundamental regulator of host metabolism and of innate and adaptive immunity. Its bacterial composition but also the synthesis of multiple molecules resulting from bacterial metabolism vary according to diet, antibiotics, drugs used, and exposure to pollutants and infectious agents. Microbiota modifications induced by recent changes in the human environment thus seem to be a major factor in the current epidemic of metabolic/inflammatory diseases (diabetes mellitus, liver diseases, inflammatory bowel disease, obesity, and dyslipidemia). Epidemiological and preclinical studies report associations between bacterial communities and cholesterolemia. However, such an association remains poorly investigated and characterized. The objectives of this review are to present the current knowledge on and potential mechanisms underlying the host-microbiota dialogue for a better understanding of the contribution of microbial communities to the regulation of cholesterol homeostasis.

## Introduction

Atherosclerosis is the underlying cause of the majority of cardiovascular disease (CVD) events, the complications of which can be fatal (myocardial infarction, sudden death, and ischemic cerebral accidents). According to WHO projections, exposure to multiple genetic and environmental risk factors and the growing number of dysmetabolic conditions (metabolic syndrome, type 2 diabetes mellitus, obesity, non-alcoholic fatty liver diseases) will contribute to making atherosclerotic cardiovascular disease (ACVD) a leading cause of death in the world by 2030 (Kaptoge et al., 2019). Among the etiological factors of this multifactorial pathology, circulating levels of total cholesterol (TC) or LDL-Cholesterol (LDL-C) represent major risk factors for ACVD. Consistent evidence from numerous epidemiological, clinical, and genetic studies unequivocally establishes a causal role of LDL-C in ACVD (Ference et al., 2017). A major instigating event is the recognition of oxidized-LDL-C by immune cells due to molecular mimicry with foreign antigens, thus promoting chronic inflammatory and self-perpetuating responses (Wolf and Ley, 2019).

Genetics plays an important role in regulating the levels of TC and associated lipoproteins (van Dongen et al., 2013; Hoffmann et al., 2018), yet genetic variation may account for 20% of plasma cholesterol levels (Surakka et al., 2015; Hoffmann et al., 2018). Environmental factors such as the amount and composition of the diet (Mente et al., 2017) as well as dietary cholesterol intake (Griffin and Lichtenstein, 2013) are well-established contributors; however, the individual-level contribution of intestinal microbiota to cholesterol homeostasis and the relevant pathways through which microbiota may exert their actions need to be documented and characterized. The gut microbiota functions as an endocrine system, which communicates with distal organs through metabolic pathways (Cani and Knauf, 2016). Additionally, modifications in the gut microbial ecosystem induced by external factors may cause radical changes in the symbiotic relationship between the microbiota and the host, and thus contribute to the low-grade inflammation that is constitutive of metabolic diseases (Cani et al., 2007; Fändriks, 2017). As a consequence, these modifications may account for a substantial proportion of the variation of plasma lipids, including cholesterol levels (Fu et al., 2015). In this context, the objectives of this review are to explore pre-clinical and clinical evidence and mechanisms linking gut microbiota and host-cholesterol metabolism in conditions of normal or altered homeostasis.

## Classical Risk Factors Associated With Cholesterolemia

Genome-wide association studies (GWAS) have identified multiple human genetic variants contributing to plasma LDL-C and TC concentrations (Willer et al., 2013; Surakka et al., 2015; Liu et al., 2017; Hoffmann et al., 2018). These latter studies identified 289 and 189 independent variants significantly associated with circulating levels of TC and LDL-C, respectively (Willer et al., 2013; Liu et al., 2017). These genetic polymorphisms collectively account for the phenotypic variance of nearly 20% of TC and LDL-C (Surakka et al., 2015; Hoffmann et al., 2018). Among them, only 1.7 to 2.5% of subjects with elevated LDL-C levels were carrying the known genetic variants identified from familial hypercholesterolemia (LDLR, APOB, and PCSK9) (Abul-Husn et al., 2016; Khera et al., 2016). The results of both twin and family studies estimated a heritability of 46-57% for TC and LDL-C (Yu et al., 2005; Goode et al., 2007; van Dongen et al., 2013). In this respect, we can estimate that environmental/lifestyle factors may account for not less than 50% and up to 80% of the complementary fluctuations of TC and LDL-C. Moreover, development of atherosclerosis and regulation of plasma TC and LDL-C levels are also closely linked to consumption of dietary fatty acids, dietary fibers, carbohydrates, and alcohol, as well as to obesity, tobacco use, and level of physical activity (Yusuf et al., 2004); most of these CV risk factors are correlated with significant changes in the gut microbial ecosystem (Cerdá et al., 2016; Costantini et al., 2017; Savin et al., 2018; Medina et al., 2019; Sarin et al., 2019). Among the best known, saturated-fatty acids, trans-fatty acids, and fibers are the nutritional factors that have the most significant impact on LDL-C (Fernandez, 2001; Riccioni et al., 2012).

Reduction in body weight in severely obese subjects has a modest influence on TC and LDL-C, with each kilogram lost associated with a decrease of ∼0.8 mg/dL in LDL-C. When weight reduction is even higher (e.g., bariatric surgery), the cholesterol-lowering effect is even more pronounced (Benetti et al., 2013). LDL-C can be reduced by regular physical activity (Leon and Sanchez, 2001), as suggested by new genetic variants interacting with physical activity and associated with cholesterol levels (Kilpeläinen et al., 2019). Additionally, other common causes of elevated LDL-C such as biliary obstruction, nephrotic syndrome, hypothyroidism, and pregnancy (Stone et al., 2014) have been connected to adverse effects on the gut microbiota composition (Ejtahed et al., 1969; Tsuji et al., 2018; Lv et al., 2019; Vieira-Silva et al., 2019).

## The New Player: Commensal Gut Microbiota

In the last decade, research developments have positioned the commensal gut microbiota at the interface between living organisms and the environment and demonstrated its considerable influence on optimum metabolic functioning (Sekirov et al., 2010). One of the contributions of gut bacteria to host biology is the circulating pool of bacteria-derived metabolites (Nicholson et al., 2012), which can reach or exceed concentrations achieved by a typical drug dose (μM to mM). In many cases, these co-metabolites signal through specific receptors and impact multiple metabolic pathways and host biology. Nearly half of the circulating metabolites are believed to come from bacterial metabolism (Wikoff et al., 2009; Sridharan et al., 2014). In the symbiotic relationships established between resident microorganisms and the host, bacteria benefit from a stable environment (nutrients, temperature, pH, osmolarity, oxygen pressure), and the biological functions of the microbiota are increasingly seen as essential to health: maturation of the immune system, metabolic and nutritional functions, and protection against pathogens. A growing number of pathologies are associated with combined quantitative and qualitative dysbiotic changes in the intestinal microbiota composition and function: diabetes, obesity, cancer, inflammatory bowel disease, autoimmune and allergic diseases, autism spectrum disorders, anxiety, and depression (Lynch and Pedersen, 2016). The microbiota thus appears to be a critical player at the crossroads of physiology and multiple pathologies. It is also emerging as a powerful transmission channel of environmental changes linked to diet and exposure to drugs, antibiotics, pollutants, and infectious agents. Microbiota modifications induced by recent changes in the human environment thus seem to be a determining factor in the current epidemic of chronic metabolic and inflammatory diseases.

More than 65 million years of mammalian-microbe co-evolution has led to an interdependence. The diversity of bacterial genes allows a wide variety of metabolic activities, such as energy extraction (5–10% of the daily energy requirements of the host) by digesting macromolecular complexes (polysaccharides, glycosaminoglycans, glycoproteins) that are not easily digestible by humans. Bacterial genes also allow the synthesis of vitamins (Yoshii et al., 2019), neurotransmitters (Onaolapo et al., 2020), and metabolites derived from tryptophan (Agus et al., 2018); they can also provide substrates that can feed critical metabolic pathways of the host (short-chain fatty acids) (Sun et al., 2017); they metabolize steroids such as cholesterol (Allayee and Hazen, 2015) or its derivatives, for instance, bile acids (Ridlon et al., 2014) and can thus influence the metabolism of lipids and cholesterol of the host. They can also contribute to or suppress the detoxification of xenobiotics and the biological activities of drugs (Koppel et al., 2017). In this ecosystem, eukaryotic and prokaryotic genes will constitute a reservoir of metabolic response that can be mobilized as a function of nutritional and xenobiotic intakes (Foster et al., 2017).

However, the complexity of intestinal microbial communities and their dialogue with the host’s metabolic pathways make functional connections complicated to disentangle in these pathologies. The fundamental challenge now is to understand the causal dimension of these relationships.

## Overview of the Epidemiology of the Gut Microbiota-Cholesterolemia Relationship

Recent data from epidemiological studies report associations between phylum, bacterial taxa, and cholesterolemia (Koren et al., 2011; Karlsson et al., 2013; Le Chatelier et al., 2013; Fu et al., 2015) (**Table 1**). These data are based on microbial taxonomy derived from 16S rRNA gene sequencing (Karlsson et al., 2013; Fu et al., 2015) or whole-genome shotgun sequencing of microbial genes collectively present in feces (Karlsson et al., 2013; Le Chatelier et al., 2013), methods that reflect the current state of the art. However, these repertoires of genes or bacterial species do not make it possible to directly report microbial functions, which can vary considerably from one strain to another within the same species. Besides, the repertoire of genes identified at the bacterial DNA level does not necessarily reflect the repertoire of functions that can or will be expressed in the host. Nevertheless, cross-validation analysis on fecal taxonomy and on circulating lipid and lipoprotein levels from 893 individuals of the general Dutch population support a contribution of the microbiome to 1.5% of the variance in TC and 0.7% in LDL-C regardless of age, gender, and genetics, with the family of *Clostridiaceae/Lachnospiracease* *families* being specifically associated with LDL-C (Fu et al., 2015). Comparable results are found by whole-genome analysis approaches on the same cohort enlarged up to 1135 individuals (Zhernakova et al., 2016). Of note, this population is primarily composed of normolipidemic subjects displaying a mean TC and LDL-C of 1.97 ± 0.39g/L and 1.24 ± 0.36g/L, respectively. These convergent data indicate that circulating concentrations of TC and LDL-C are correlated with changes in microbiota composition, and a recent study conducted on the LifeLines-DEEP cohort (1293 subjects) supports this hypothesis. In this study, 92 plasma proteins associated with CV risk were quantified. Among them, the variance in the concentration of circulating LDL receptor is explained by microbial factors for 5%, while only 0.1% is explained by genetic factors (quantitative trait locus) (Zhernakova et al., 2018).

**Table 1:** List of major clinical evidence.

Evidence	Cohort	Correlation Association	References
**Epidemiological**	268 healthy subjects (16S)	Enterotypes/ Cholesterol	(de Moraes et al., 2017)
	896 healthy subjects (16S)	Phylum/ Cholesterol	(Fu et al., 2015)
	1135 healthy subjects (MGS)	Taxa/ Cholesterol	(Zhernakova et al., 2016)
	Metabolic Syndrome (MGS)	Fecal microbial gene richness and diversity/Cholesterol	(Karlsson et al., 2013) (Le Chatelier et al., 2013)
	Dyslipidemic cohort	nd	nd
**Dietary intervention**	49 overweight/ obese adults (MGS)	Fecal microbial gene richness/ LDL-C	(Cotillard et al., 2013) (Dao et al., 2016)

nd, not done.

In patients with metabolic syndrome, interindividual variations in circulating TC and LDL-C are associated with microbial gene richness and diversity (Le Chatelier et al., 2013). The correction of diversity loss after nutritional intervention in dysmetabolic patients corrects hypercholesterolemia (Cotillard et al., 2013) and is associated with a higher abundance of *Akkermansia muciniphila* (Dao et al., 2016) (**Table 1**). When hypercholesterolemia coexists with obesity, hypertension, and glucose intolerance, it should be taken into account that multiple mechanisms can contribute to the regulation of cholesterolemia, including pathways through which these pathologies are associated collectively (metabolic syndrome) or individually with dysbiosis (Ussar et al., 2015; Lim et al., 2017; Hoyles et al., 2018). Finally, a study in patients displaying clinical features of atherosclerosis in comparison with control subjects found associations between TC, LDL-C, and the oral abundance of some bacterial species (Koren et al., 2011).

Interestingly, studies performed in pigs, which have a metabolism and microbiome much closer to humans than rodent models, showed a significant contribution of the caecal microbiome of 5.6% to TC and of 2.8% to LDL-C (Huang et al., 2017). Additionally, most microbial taxa positively associated with TC and LDL-C belong to the pathogenic bacteria. These data are consistent with the known relationship between inflammation and serum cholesterol (Khovidhunkit et al., 2004), which needs to be further explored.

Although obtained in general populations, the influence of the gut microbiota on cholesterol levels would undoubtedly benefit from an investigation in a dyslipidemic cohort where reciprocal effects of hypercholesterolemia on microbiota functions may amplify dysbiosis and its consequences on host metabolism. Indeed, such correlative data do not establish a causal link. A disease may modify the gut microbiota, and conversely, the gut microbiota may trigger or aggravate a condition. Additionally, the bacterial species distribution is not homogeneous along the digestive tract, and fecal microbiota mostly reflects colonic species. Thus, feces analysis neglects the potential involvement of commensal species of the small intestine in dysbiosis, though this represents an essential site for the metabolism of cholesterol. Therefore, evaluation of the contribution of the microbiota to cholesterol levels is not optimal. In a recent study, albeit in a small cohort, the authors show that in hyperlipidemic patients, the higher prevalence of small intestinal bacterial overgrowth (SIBO) is positively associated with LDL-C levels (Kvit et al., 2019).

## Modulation of the Microbiota and Its Impact on Cholesterolemia in Humans

After birth primo-colonization of the digestive tract, the gut microbiota becomes richer and more diversified all through life as a result of environmental challenges such as those from nutritional status, cultural habits, and drug treatments (Rothschild et al., 2018). Thus, the effect of changes in diet composition, eating patterns, on cholesterolemia is likely related to the benefits of prebiotics (Beserra et al., 2015) or a Mediterranean (Filippis et al., 2016) or vegan (versus omnivorous) (Wu et al., 2016) diet, which correlate with variations in microbiota composition. Likewise, lipids are strongly modified at birth and at weaning, two periods associated with major changes in microbial composition (Nuriel-Ohayon et al., 2016) and bile acid (BA) metabolism (Jönsson et al., 1995), which can influence circulating lipid and lipoprotein concentrations (Joyce et al., 2014). When administered orally, antibiotics induce a reduction in circulating cholesterol, which is strongly associated with changes in the composition of microbiota-derived secondary BAs (Samuel and Whithe, 1961; Samuel et al., 1973; Miettinen, 1979).

## Gut Microbiota and Cholesterol Traits in Preclinical Models

Numerous studies that specifically evaluated the potential role of the microbiota in the regulation of cholesterol homeostasis have been conducted by using conventional approaches to eradicate the microbiota by either antibiotic therapy or an axenization procedure. These latter studies revealed that the absence of microbiota significantly influences cholesterolemia (**Table 2**). However, these associations are not all consistent in the normolipidemic context. Some teams show decreases in TC (Rabot et al., 2010) (Velagapudi et al., 2010; Joyce et al., 2014; Zhong et al., 2015), while others find no effect (Danielsson and Gustafsson, 1959; Sayin et al., 2013; Out et al., 2015; Caesar et al., 2016; Mistry et al., 2017; Zarrinpar et al., 2018) or even an increase (Danielsson and Gustafsson, 1959; Caesar et al., 2016). The potential underlying explanations of such variability involve (i) differences in microbiota composition between the animal facilities, (ii) normolipidemic wild type mice carry the majority of plasma cholesterol in HDL, (iii) reduced penetrance of the influence of the microbiota in a homeostatic context, as observed in the general human population (Fu et al., 2015). By contrast, in a commonly used mouse model of dyslipidemia (apolipoprotein-E and Ldl-r deficient mice), almost all studies show increased cholesterol levels in the absence of gut-microbiota (**Table 2**) (Wright et al., 2000; Stepankova et al., 2010; Chen et al., 2016; Kasahara et al., 2017; Lindskog Jonsson et al., 2018; Kiouptsi et al., 2019; Le Roy et al., 2019). The lipid-rich environment is associated with an impoverishment of gut microbiota diversity and richness (Martínez et al., 2013; Bo et al., 2017; Tran et al., 2019), increased intestinal barrier permeability, and endotoxemia (Netto Candido et al., 2018; Schoeler and Caesar, 2019; Wisniewski et al., 2019). Thus, in this inflammatory context, the influence of microbiota on cholesterolemia is revealed, as suggested by data obtained in the pig model (Huang et al., 2017). These findings are in agreement with data obtained in rodent models in which hypercholesterolemia associated with acute activation of innate immune receptors by endotoxin/lipopolysaccharide (LPS) is connected with an increase hepatic cholesterol synthesis and VLDL production and decreased VLDL and LDL clearance (also termed the lipemia of sepsis) (Harris et al., 2000). The underlying molecular mechanisms involve decreased nuclear receptor signaling of peroxisome proliferator-activated receptor (PPAR), liver X receptor (LXR), farnesoid X receptor (FXR), and retinoid X receptor (RXR) (Khovidhunkit et al., 2004). It additionally involves inhibition of reverse cholesterol transport (RCT) at multiple points, including decreased hepatic production of apolipoprotein A1, cholesterol ester transfer protein (CETP), ATP binding cassette transporters ABCG5 and ABCG8, and Cyp7a1. These findings are consistent with the association found of the lipopolysaccharide receptor Toll-like receptor 4 (TLR4) and NIMA‐related kinase 7 (NEK7) polymorphisms with LDL-C in human (Zhu et al., 2015; Gomes Torres et al., 2019). NEK7 is a serine/threonine kinase required for NLRP3 (NOD-, LRR- and pyrin domain-containing protein 3) inflammasome assembly. However, this relationship should be tempered as chronic TLR-signaling deficiency in MyD88^-/-^/Apoe^-/-^ (Björkbacka et al., 2004) (Michelsen et al., 2004), TLR4^-/-^/Ldl-r^-/-^, (Ferreira et al., 2015), TLR2^-/-^/Apoe^-/-^, and TLR4^-/-^/Apoe^-/-^ (Higashimori et al., 2011) mice is not associated with changes in cholesterol levels as compared to control mice. It should also be noted that the evidence for the role of microbiota in genetically modified mice is difficult to interpret, as numerous studies in the literature do not report the experimental conditions (production of experimental groups, use of littermates, housing conditions). Indeed, fecal microbiota is partially normalized by extended co-housing conditions, due to coprophagic and grooming behaviors, thus abrogating microbiota-genotype dependent phenotype.

**Table 2:** List of major pre-clinical evidence.

Model	Diet	Phenotype in germ-free	References
**Axenic Normolipidemic** **Rat**	CD	Chol 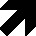	(Danielsson and Gustafsson, 1959)
	CD with 0.5% cholesterol	Chol 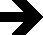	(Danielsson and Gustafsson, 1959)
**Axenic** **Normolipidemic** **Mice**	HFD 0.03% cholesterol	Chol 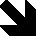	(Rabot et al., 2010)
	CD*	Chol 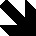	(Velagapudi et al., 2010)
	CD	Chol 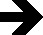	(Sayin et al., 2013)
	WD with 0.2% cholesterol	Chol 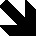	(Zhong et al., 2015)
	CD source of fat: lard	Chol 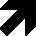	(Caesar et al., 2016)
	CD source of fat: fish oil	Chol 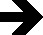	(Caesar et al., 2016)
	CD	Chol 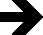	(Mistry et al., 2017)
**Normolipidemic** **Mice** **(axenization by** **a mixture of antibiotics)**	CD*	Chol 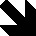	(Joyce et al., 2014)
	CD	Chol 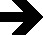	(Out et al., 2015)
	CD	Chol 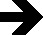	(Zarrinpar et al., 2018)
	CD	Chol 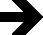	Personal observations
**Axenic** **Dyslipidemic** **Mice**	Apoe^-/-^ 0.15% cholesterol diet	Chol 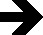	Wright et al., JEM 2000
	Apoe^-/-^ CD	Chol 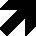	(Stepankova et al., 2010)
	Apoe^-/-^ 2% cholesterol	Chol 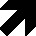	(Stepankova et al., 2010)
	Apoe^-/-^ CD	Chol 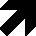	(Kasahara et al., 2017)
	Apoe^-/-^ CD	Chol 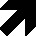	(Lindskog Jonsson et al., 2018)
	Apoe^-/-^ WD	Chol 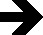	(Lindskog Jonsson et al., 2018)
	Ldlr^-/-^ CD	Chol 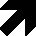	(Kiouptsi et al., 2019)
	Ldlr^-/-^ 0.2% cholesterol diet	Chol 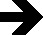	(Kiouptsi et al., 2019)
	Apoe^-/-^ CD	Chol 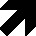	(Chen et al., 2016)
**Dyslipidemic** **Mice** **(axenization by** **a mixture of antibiotics)**	Apoe^-/-^ 0.15% cholesterol diet	Chol 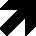	(Chen et al., 2016)
	Apoe^-/-^ CD	Chol 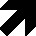	(Le Roy et al., 2019)
	Ldlr^-/-^ CD	Chol 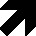	(Le Roy et al., 2019)
**Dyslipidemic** **Mice** **FMT**	Apoe^-/-^ CD	Cholesterol levels transmitted**	(Le Roy et al., 2019)

All studies involved mice of C57Bl6 genetic background, except for two studies that used Swiss Webster mice (indicated by *). CD, chow diet; HFD, high-fat diet; WD, western diet; FMT, fecal material transfer.

**Phenotype in recipient mice.

↗Increased; ↘decreased; →no changes.

Finally, using a standardized method, our recent work (Le Roy et al., 2019) demonstrates the microbiota-dependent transmissibility of a significant proportion of the cholesterol level (around 15-20%). Indeed, transplantation of the microbiota from hypercholesterolemic (without known genetic cause) human donors into recipient mice is sufficient to transfer the phenotype compared to the same experiment performed with normolipidemic donors. The more hypercholesterolemic phenotype is associated with “low hepatic cholesterol synthesis” and “high intestinal cholesterol absorption” traits in recipient mice. Several bacterial phylotypes affiliated with *Beta-proteobacteria phylum*, *Alistipes genus, and Barnesiella genus* were enriched in hypercholesterolemic mouse recipients. Similarly, *Alistipes* were recently associated with TC and LDL-C in HFD-fed hamsters treated with a chitin9-derived polysaccharide (chitosan) (Tong et al., 2019).

It is also of particular clinical interest to show the influence of the intestinal microbiota on the balance between absorption and cholesterol synthesis (Le Roy et al., 2019) since it has been observed in human cohorts that “high absorption” and “low synthesis” patterns are associated with higher LDL-C levels and are predictive of cardiovascular events (Matthan et al., 2009; Silbernagel et al., 2010; Weingärtner et al., 2011). Thus, inter-individual evaluation of microbiota diversity or dysbiosis opened up new opportunities for better therapeutic decision-making in ASCVD.

## Gut Bacterial Metabolites, the New Frontier for Defining Pathological Metabolotypes

### Primary Bile Acids

**Figure 1:**
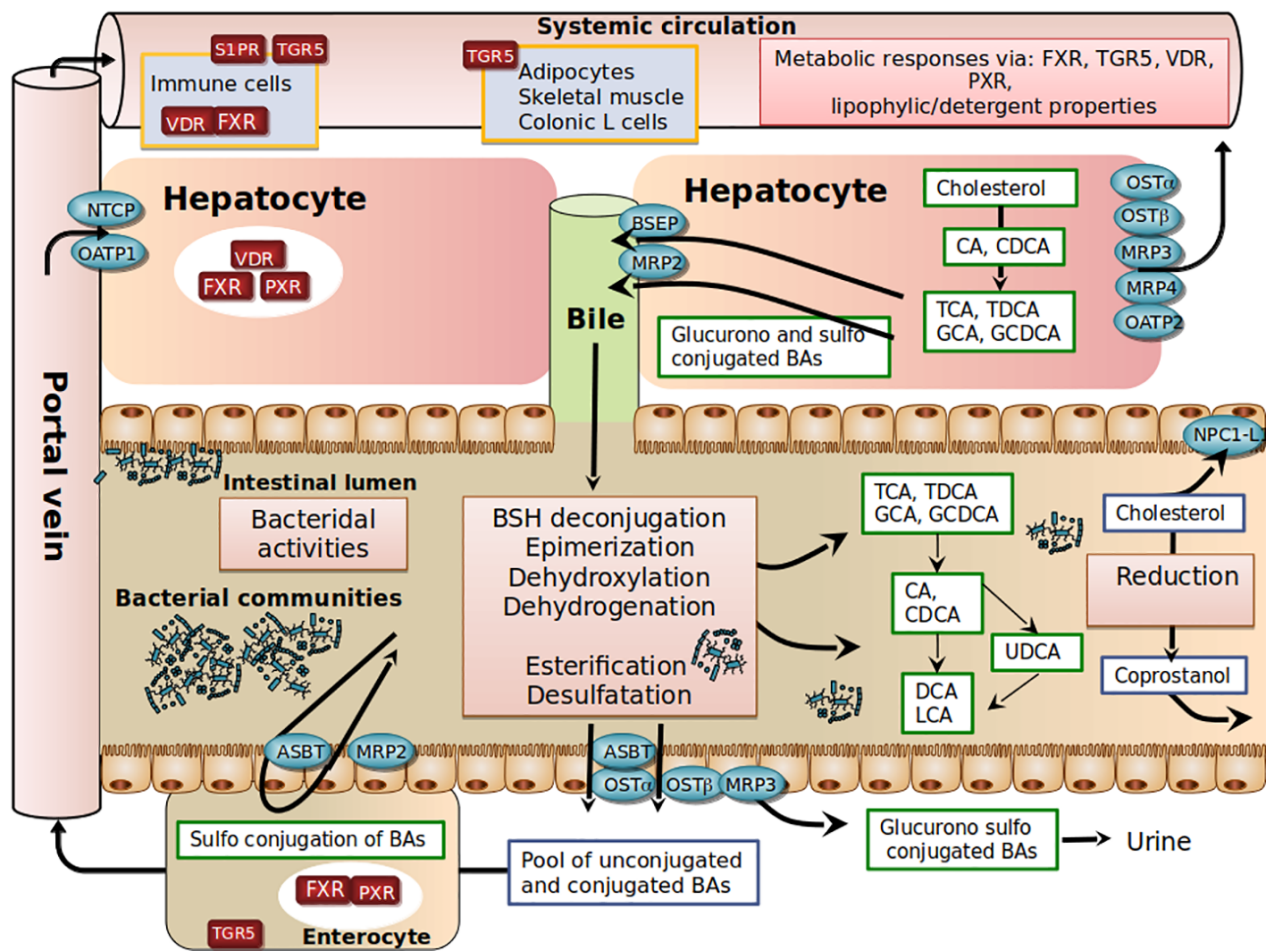
Schematic view of host-gut microbial co-metabolism of bile acids and cholesterol in enterohepatic circulation.

Bile acid (BA) biosynthesis is the predominant metabolic pathway for cholesterol catabolism in the human body. The conversion of cholesterol to bile acids is a process performed by a set of hepatic enzymes necessary for the conversion of the steroid nucleus of cholesterol, the elimination of the side chain, amidation on the side chain with either glycine or taurine (GCA, GCDA, TCA, TCDCA) and eventually sulfonation or glucuronidation in the steroid backbone (Hofmann and Hagey, 2014). Another critical aspect of BA physiology is their circulation in the enterohepatic cycle, a finely tuned and orchestrated system, in which BAs synthesized in the liver are actively transported in the bile ducts, stored in the gallbladder, then secreted in the duodenum, absorbed again in the ileum, and recaptured by the liver *via* the portal circulation. Each stage of this enterohepatic cycle is influenced by diet, hormonal cross-regulation, and bacterial activities that maintain a functional and non-toxic supply of bile acids in circulation. Indeed, BAs have a pro-inflammatory and cytotoxic potential when they are not regulated, due to their detergent activity and destabilization of membranes, as shown in cholestatic liver diseases (**Figure 1**).

In the post-prandial period, primary BAs are released into the intestinal lumen. Due to their amphiphilic properties, primary BAs adsorb at an oil–water interface to form mixed micelles with other bile lipids (cholesterol, phosphatidylcholine), fat-soluble vitamins (such as vitamins A, D, E, and K) and lipolysis products (free fatty acids, 2-monoglycerides). In the absence of bile secretion, fat absorption is impaired (Hofmann, 1999; Dawson and Karpen, 2015). In the ileum, a highly efficient transporter system allows active reabsorption of conjugated-BAs redirected to the liver through mesenteric and hepatic portal veins (Dawson and Karpen, 2015). At each enterohepatic cycle (4-5 cycles per day), about 5% of the non-absorbed BAs are released into the colon, modified by bacteria, and then excreted. This represents around 600 mg per day, the loss of which is compensated for by an equivalent synthesis from hepatic cholesterol. The co-excretion of fecal sterols and BAs in a 2:1 ratio in humans therefore represents a significant pathway for regulating cholesterol homeostasis (Groen et al., 2014).

### Secondary Bile Acids

Another complexity in BA metabolism is the modification of the BA structure by intestinal bacteria (Ridlon et al., 2014). BAs that are not reabsorbed encounter anaerobic resident bacteria in the colon (**Figure 1**). Microbial enzymes such as bile salt hydrolases (BSH) deconjugate conjugated -BAs, bacterial 7 α-dehydroxylases and 7 β-dehydroxylases convert CA and CDCA to deoxycholic acid (DCA) and lithocholic acid (LCA), respectively. Bacterial 7β-isomerization of the 7α-hydroxyl group of CDCA forms ursodeoxycholic acid (UDCA). Sulfated and glucuronidated BAs formed during hepatic detoxification to facilitate their urinary and fecal excretion can be hydrolyzed of their ester linkage by microbial enzymes (Takikawa et al., 1983; Gérard, 2013), ultimately leading to the presence of a vast repertoire of secondary BAs. In the colon, unconjugated BAs produced by microbial metabolism can diffuse passively over the intestinal border and can eventually be captured by the liver through multispecific organic anion-transporting polypeptide (OATP) transporters that can vehicle unconjugated BAs (Unconj-BA) and sulfated BAs (Dawson and Karpen, 2015). In the liver, conjugated BAs are more efficiently recycled from portal blood at the hepatic basolateral membrane by the high-affinity sodium-dependent taurocholate cotransporting polypeptide (NTCP) than are unconjugated BAs (Angelin et al., 1982; Hofmann and Hagey, 2014; Eggink et al., 2018). The estimated hepatic fractional uptake of total BAs ranges from 50 to 90% depending on the bile acid structure (Angelin et al., 1982) and is reflected by differences in systemic blood concentration versus portal blood concentration (Eggink et al., 2018). During the completion of the enterohepatic cycle, unamidated BAs can be conjugated again in the liver, leading to the formation of their glycine or taurine conjugates (GDCA, GLCA, GUDCA, TDCA, TLCA, and TUDCA), while CDCA and LCA can be 6α-hydroxylated to form hyocholic acid (HCA) and hyodeoxycholic acid (HDCA), respectively (Bodin et al., 2005). UDCA represents about 4% of total fecal BAs, and a cholesterol-lowering effect has been reported in patients with primary biliary cirrhosis (Poupon et al., 1993) or hypercholesterolemia (Cabezas Gelabert, 2004). Other exclusively microbial activities, including esterification, oxidation, and desulfation, contribute to the high chemical diversity and changes in hydrophobicity (Ridlon et al., 2014; Devlin and Fischbach, 2015; Watanabe et al., 2017) and bile acid signaling activities (Thomas et al., 2008; de Boer et al., 2018) (**Figure 1**).

It should be noted that molecular species of BAs are involved not only in lipid metabolism but also in carbohydrate metabolism, energy homeostasis, and host immune responses through their agonistic or antagonistic activities on diverse receptors, the best characterized being the farnesoid X nuclear receptor (FXR) and the TGR5 membrane receptor (Thomas et al., 2008; de Boer et al., 2018); these aspects have been extensively reviewed elsewhere (Houten et al., 2006; Thomas et al., 2008; Lefebvre et al., 2009). Basically, FXR impacts cholesterolemia through the repression of CYP7A1, the rate-limiting enzyme that catabolizes conversion of cholesterol into BAs, resulting in decreased hepatic cholesterol content, followed by upregulation of the LDL-receptor expression and activity, which consequently reduces plasma LDL-C levels. This mechanism underlies the hypocholesterolemic effect of BAs sequestrants (Spinelli et al., 2016). Administration of obeticholic acid (FXR agonist) to chow-fed mice elevates liver LDL receptor expression by mRNA stabilization and reduces plasma LDL-C in mice (Singh et al., 2018). Of note, a recent study discovered a novel association of a variant in human NR1H4 gene (encoding the BA receptor FXR) with levels of TC and LDL-C (Deaton et al., 2018), thus highlighting the role of FXR in the regulation of plasma cholesterol levels in humans.

In the context of cholesterol reduction following antibiotic treatment, in humans (Jenkins et al., 2005), the prevailing hypothesis is that inhibition of the conversion of primary BAs to secondary BAs reduces their hydrophobicity, which results in poorer reabsorption by passive diffusion through the colonic epithelium. Similarly, this decrease in hydrophobicity of BAs is associated with a poorer micellization of cholesterol, which would, therefore, be less efficiently absorbed. These joint activities contribute to the outflow of BAs and cholesterol in the stool and therefore to a decreased sterol pool of the whole body. However, in humans, other molecular mechanisms must coexist to the extent that treatments with primary bile acids (CDCA) or secondary bile acid (LCA) only slightly alter the absorption of cholesterol and the serum concentrations of LDL-C (Wang et al., 2006). Other putative mechanisms qualitatively and quantitatively modulating the pool of bile acids may be at work; a pool of depleted BAs will be associated with the proliferation of pro-inflammatory microbes (Kakiyama et al., 2013) and intestinal barrier dysfunction (Kang et al., 2017), and a pool reconstituted after transfer of fecal material or liver transplantation will correct endotoxemia (Kang et al., 2017; Bajaj et al., 2018) and lipidemia (Kang et al., 2017).

Interestingly, the treatment of 51 naive type-2 diabetic patients with an antidiabetic (acarbose: a tetra-saccharide inhibiting hydrolysis of carbohydrates in the upper intestine and thus reducing glucose absorption) led to improvements of glycemia and cholesterolemia (Gu et al., 2017). These changes were correlated with variations in plasma BA profiles. The primary-BA/secondary-BA ratio and UDCA and T-DCA concentrations were negatively correlated with plasma cholesterol. Accordingly, metagenomics analysis confirmed a lower capacity for 7α/β dehydroxylation of BAs after acarbose treatment. The relative abundances of baiE (rate-limiting enzyme for 7α-dehydroxylation) and baiI (7β-dehydratase) were significantly decreased after acarbose treatment. Phylogenic analysis established a strong inverse correlation between *Lactobacillus rhamnosus* and plasma cholesterol and LDL-C levels. Interestingly, decreases in plasma cholesterol levels associated with acarbose treatment were associated with a decline in *allistipes* spp., in accordance with recent studies (Le Roy et al., 2019; Tong et al., 2019).

In conclusion, the mechanisms underlying bile acid-cholesterol-lowering relationships remain largely undefined. The specific roles of bile acids *in vivo* remain difficult to disentangle, due to the large number of compounds and biological properties involved, including detergent and bactericidal activities and FXR signaling potential (**Figure 1**). The picture is even more complex if we consider the newly involved receptors such as pregnane X receptor (PXR), vitamin D3 receptor (VDR), muscarinic acetylcholine receptors, and sphingosine 1-phosphate receptor (S1PR). In addition to qualitative parameters, it is also necessary to consider all the poorly known circadian and post-prandial quantitative variations of BAs that will need to be well-defined to better understand the impact of BAs on cholesterol homeostasis (Han et al., 2015).

### Short-Chain Fatty Acids (SCFAs)

SCFAs are the main end-product produced by the bacterial fermentation of non-digestible dietary fibers in the caecum and proximal colon. Consumption of dietary fibers such as inulin, oat bran, and pectin is effectively associated with lower plasma cholesterol levels, with reductions in cholesterol level ranging from 0.5% to 2% per gram of intake (Ripsin et al., 1992). Fibers reduce both TC and LDL-C (Anderson and Chen, 1979) through increased BA excretion and decreased hepatic synthesis of cholesterol (Vahouny et al., 1980; Jenkins et al., 2010). Other potential mechanisms are related to the microbiota-dependent formation of SCFAs (acetate, propionate, butyrate) that are produced and can be used as a macronutrient source of energy. Alternatively, SCFAs can act as hormone-like signaling, entering the portal circulation to ultimately bind to G-protein-coupled receptors (GPR) in numerous cells (Maslowski et al., 2009) and inhibit the histone deacetylase (HDAC), resulting in numerous epigenetic modifications in targeted cells (Riggs et al., 1977; Candido, 1978).

Several studies have described the role of SCFAs on immunity (Aguilar et al., 2014) and its protective effects against cardiovascular disease (Bazzano et al., 2003; Fernandez et al., 2019), yet few of them have individually examined the effects on cholesterol levels. *Ex vivo* and *in vivo* studies have shown that acetate and butyrate (but not propionate) are potential precursors of cholesterol synthesis that can be incorporated into the endogenous cholesterol synthesis pathway (Wolever et al., 1991; Demigné et al., 1995; den Besten et al., 2013). By contrast, earlier studies reported inhibition of cholesterol synthesis by propionate through decreased expression of the HMGCS and HMGCR genes (Bush and Milligan, 1971; Rodwell et al., 1976; Demigné et al., 1995), white more recent studies have not confirmed such effects (Zhao et al., 2017). Accordingly, in healthy subjects, the oral administration of propionate does not lower plasma cholesterol but does increase HDL and triglycerides levels (Venter et al., 1990). In vivo, supplementation of a cholesterol-rich diet with acetate in rats resulted in a lower increase of TC levels associated with lower hepatic HMGCS and increased conversion of cholesterol into BAs due to the upregulation of Cyp7a (Fushimi et al., 2006). A similar rise in Cyp7a was observed in Apoe-deficient mice fed with a cholesterol-rich diet supplemented with butyrate, with additional beneficial effects on the “reverse cholesterol transport” (RCT) (Du et al., 2019). The connection between SCFAs and BAs metabolism was further reported in Syrian hamsters (Zhao et al., 2017). The addition of acetate, propionate, or butyrate to a cholesterol-rich diet resulted in decreased CT levels and LDL-C/HDL-C ratio and to increased fecal excretion of BAs (LCA, DCA, CDCA, CA). Expression of the SREBP2, LDLR, and CYP7A1 genes was also involved (Zhao et al., 2017). The effects of SCFAs were more specifically addressed in mice deficient for FFAR2/GPR43, one of the SCFA receptors (Bjursell et al., 2011). High-fat fed GPR43-deficient mice displayed lower CT levels than control mice.

**Figure 2:**
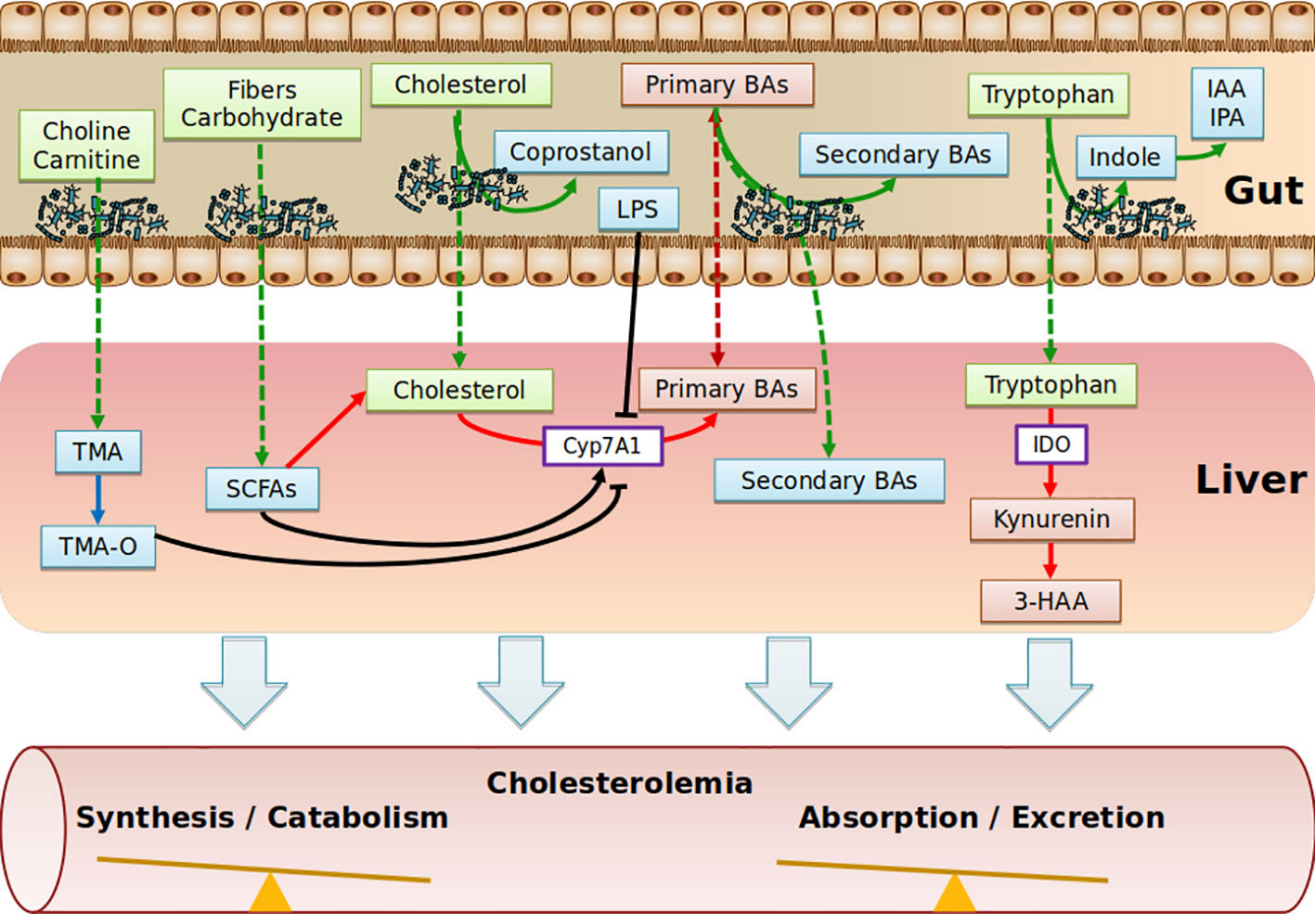
Schematic depicting the range of putative pathways through which the gut metabolites impact on cholesterol metabolism. Diet nutriments (green boxes) are metabolized and transformed into microbial metabolites (blue boxes) by gut microbiota. Purple boxes represent enzymes. Red boxes represent host metabolites. TMA, Trimethylamine; TMA-O, Trimethylamine-Oxide; SCFAs, Short Chain Fatty Acids; BAs, Bile Acids; IAA, Indole-3-acetic acid; IPA, Indole-3-propionic acid; IDO, indoleamine2,3-dioxygenase; 3-HAA, 3-hydroxyanthranilic; LPS, lipopolysaccharides.

Collectively, the role of SCFAs in cholesterol levels is poorly defined. SCFAs may be used as precursors of cholesterol synthesis, but the overall hypocholesterolemic effect seems to be associated with the conversion of cholesterol into BAs. Translation studies into humans will be critical to move forward (**Figure 2**).

### Trimethylamine-oxide (TMA-O)

The contributing role of intestinal microbiota to ACV diseases through the production of trimethylamine-N-oxide (TMA-O, has been recently demonstrated (Randrianarisoa et al., 2016; Tang et al., 2017; Yang et al., 2019) and has been covered in recent reviews (Brown and Hazen, 2018; Yang et al., 2019). Essentially, trimethylamine-containing dietary nutrients (choline, phosphatidylcholine, γ-butyrobetaine, and carnitine) are metabolized by microbes, leading to the production of trimethylamine (TMA), which is rapidly converted by host hepatic flavin monooxygenase 3 (FMO3) into trimethylamine N-oxide (TMA-O). Interestingly, in dyslipidemic mouse models, TMA-O affects cholesterol homeostasis mainly by suppressing reverse cholesterol transport (RCT) (Koeth et al., 2013) and impacting the BA metabolic pathways at multiple levels (decreased expression of hepatic BAs synthetic enzymes (Cyp7a1 and Cyp27a1) and hepatic BA transporters (Oatp1, Oatp4, Mrp2, and Ntcp) (Koeth et al., 2013). Additionally, TMA-O appears to promote cholesterol uptake by macrophages by inducing scavenger receptors CD36 and SRA1, both of which are involved in the intracellular accumulation of modified lipoproteins (Wang et al., 2011). Likewise, under normal dietary conditions, TMA-O did not impact plasma cholesterol levels in mice deficient for FMO genes (Veeravalli et al., 2018). Identification of TMA-O receptors would be of particular interest to substantiate a potential association of TMA-O with cholesterol levels, albeit that no significant correlations between TMA-O and TC, LDL-C, even when excluding individuals taking cholesterol-lowering medications, have been observed (Li et al., 2017). This might explain why TMA-O has been demonstrated to be a prognostic marker for ACV diseases beyond traditional risk factors (Manor et al., 2018) (**Figure 2**).

### Diet-Associated Tryptophan (Trp) Derivatives

Tryptophan is an essential amino acid that is degraded through the kynurenine pathway, leading to the generation of several biologically active compounds. Endogenous kynurenine metabolites contribute to the initiation of ACV disease. In human atherosclerotic plaques, Trp metabolites were found to be associated with unstable plaque phenotype (Taleb, 2019). Tryptophan is processed 95% by the kynurenine pathway (gut-microbiota independent) and 5% by the indole pathway (gut microbiota-dependent). Regarding the kynurenin pathway, supplementation of 3-hydroxyanthranilic (3-HAA), a tryptophan-derivative metabolite from the kynurenine pathway has anti-atherosclerotic effects, associated with lower plasma cholesterol levels in Ldl-r-deficient mice fed an HFD regime (Zhang et al., 2012) or a western diet (Berg et al., 2019). Correspondingly, indoleamine 2,3-dioxygenase (IDO) inhibition showed the exact opposite phenotype in Apoe-deficient mice fed an HFD (Polyzos et al., 2015; Liang et al., 2019), while no effect was reported in chow-diet-fed double-deficient mice for Apoe and IDO (Cole et al., 2015). IDO enzymes are involved in the catabolism of tryptophan, and the ratio of kynurenine to tryptophan (kyn/trp) can be used to reflect IDO activity. In clinical investigations, IDO activity has been reported to be positively correlated with a range of atherosclerosis risk factors in the female population, including LDL-C (Pertovaara et al., 2007). Concerning the indole pathway, tryptophan, indole-3-propionic acid, and indole-3-aldehyde were shown to be decreased in atherosclerotic patients, while kynurenine/tryptophan ratios were increased (Cason et al., 2018); still, no independent correlation with cholesterol has yet been reported. In conclusion, the scarcity of studies does not allow the indole pathway to be implicated in regulation of cholesterolemia (**Figure 2**).

## Conversion of Cholesterol Into Coprostanol

Cholesterol from the diet, bile, or intestinal cells is actively metabolized by intestinal bacteria, mainly in coprostanol (Gérard, 2013). Unlike cholesterol, coprostanol is very poorly absorbed by the intestine (Bhattacharyya, 1986). In a singular way, the rate of conversion of microbial cholesterol to coprostanol in the general human population appears to be multimodal, with an average of 65% of high converters (80% to 100% of luminal cholesterol is converted to coprostanol in the colon), 21% of intermediate converters and 14% of non-converters (Wilkins and Hackman, 1974; Midtvedt et al., 1990; Veiga et al., 2005; Benno et al., 2005). It was also demonstrated that this phenotypic characteristic was maintained in axenic rodents (without germs) colonized with a high-converter or non-converter human microbiota (Gérard et al., 2004). Finally, several clinical and preclinical studies support the hypothesis that the conversion of cholesterol in coprostanol could influence the bioavailability of cholesterol, leading to modulation of plasma cholesterol levels (Sekimoto et al., 1983; Li et al., 1995; Li et al., 1998). Larger studies are needed to validate this relationship. Notably, the disconnection between the major cholesterol uptake site (small intestine *via* the Niemann-Pick C1-Like 1 transporter (NPC1L1)) and the site of cholesterol conversion to coprostanol (colon) does not plead for a causal relationship. Nevertheless, normolipidemic subjects treated with Ezetimibe (NPC1L1 inhibitor) show residual absorption of cholesterol, which suggests as yet unidentified additional absorption mechanisms (Jakulj et al., 2016) (**Figures 1** and **2**).

## Impact of the Microbiota on Hypocholesterolemic Drug Efficacy

The gut microbiota has been shown to impact, negatively or positively, drug efficacy. This effect has been shown to result either from modifications in pharmacokinetic or pharmacodynamic properties or by synergistic/antagonistic effect of microbiota toward drugs. As a matter of fact, the impact of the microbiota on drugs is not restrained to oral drug intake, as studies have shown modifications in monoclonal antibody efficacy (Sivan et al., 2015; Doherty et al., 2018). The interaction of gut microbiota with drug efficacy/toxicity has recently been exhaustively reported upon (Spanogiannopoulos et al., 2016; Wilson and Nicholson, 2017; Clarke et al., 2019).

Direct links between the gut microbiota and hypocholesterolemic drugs are still thin. Statins, which are the leading pharmaceutical class in hyperlipemia therapeutic care, are ineffective for almost 20% of patients treated and are sometimes even deleterious (Stroes et al., 2015). Several studies have explored and demonstrated that statins can directly influence the growth and virulence of bacterial pathogens and commensal bacteria as well as combating microbial infections, such as in sepsis and pneumonia (Hennessy et al., 2016; Zimmermann et al., 2019). The first statin, “Mevastatin,” which is a metabolic product of Penicillium citrinum, was initially characterized for its antibiotic properties, and statins are now considered as adjuvant antibiotics that can impact antimicrobial resistance (Ko et al., 2017). Consequently, the role of statins deserves to be explored beyond their traditionally established indications in light of their antimicrobial potential as a regulator of gastrointestinal microbiota (Nolan et al., 2017). Only a few studies have shown the impact of the microbiota on statin efficacy. Still, these studies suggested that the microbiota participated in statin’s effect (He et al., 2017) and was responsible for statin metabolization (Yoo et al., 2014) and that the microbiota from patients unresponsive to statin was different from that of responsive patients (Sun et al., 2018).

Additional studies are required in this context and should, therefore, also be conducted on other therapeutic classes of hypolipemic drugs. Understanding the impact of the microbiota on drug efficacy/toxicity should bring us closer to personalized medicine and should result in an improvement in therapeutic care.

## Concluding Remarks

LDL-C is the primary target for the management of atherogenic dyslipidemia and the reduction of cardiovascular events. New actors such as the microbiota introduce more complexity into this multifactorial disease but allow new insight into pathogenicity and the development of new prevention and prophylaxis approaches. In addition to the usual pharmacological approaches (statins, ezetimibe, fibrates, resins, proprotein convertase subtilisin-kexin 9 (PCSK9) inhibitors), new biotherapies targeting the microbiota are possible. Indeed, the data in the literature support the notion that the microbiota has a causal contribution to the metabolism of lipoproteins and host cholesterol. The mechanisms of this reciprocal influence need to be clarified, and the advent of functional analyses of the microbiota, the development of new technologies allowing the culture of anaerobic microbes, and the advent of more and more better-performing technologies will make it possible to specify the dynamics of the relationship of the intestinal microbiota with cholesterol metabolism.

## Author Contributions

RV, PK, SB, MS, DR, MG, and PL originally conceived and wrote the manuscript. All authors read and approved the final manuscript.

## Funding

This study was supported by the Institut National de la Santé et de la Recherche Médicale (INSERM), Sorbonne Université (SU), and the Fondation de France (00029519). PK was a recipient of a research fellowship from the French Ministry of Research and Technology and from the New French Atherosclerosis Society (NSFA). MS is supported by a fellowship from the Mexican National Council of Science and Technology (CONACYT).

## Conflict of Interest

The authors declare that the research was conducted in the absence of any commercial or financial relationships that could be construed as a potential conflict of interest.
